# Compliance With Ecological Momentary Assessment Among Patients With Cancer: Systematic Review and Meta-Analysis

**DOI:** 10.2196/87506

**Published:** 2026-09-08

**Authors:** Guiyuan Ma, Cai Deng, Zitong Zhang, Qiming Ding, Can Gu

**Affiliations:** 1 Xiangya School of Nursing, Central South University, No. 172 Tongzipo Road, Yuelu District, Changsha, Hunan, 410013, China, 86 13874881548

**Keywords:** cancer, ecological momentary assessment, compliance, mobile health, meta-analysis

## Abstract

**Background:**

Patients with cancer often experience substantial fluctuations in psychological states during disease management. Traditional research tools are limited in capturing these dynamic changes in real time, constraining clinicians’ understanding of patients’ true conditions. Ecological momentary assessment (EMA) enables high-frequency, real-time data collection, providing patient-reported data with greater ecological validity. However, the effectiveness of EMA studies critically depends on patient compliance, and reported compliance rates vary widely, with a lack of systematic quantitative synthesis.

**Objective:**

This study aims to systematically review and quantitatively analyze compliance with EMA among patients with cancer, and to examine whether EMA design characteristics were associated with compliance.

**Methods:**

Web of Science, PubMed, Embase, Cochrane Library, CINAHL, PsycINFO, CNKI, and Wanfang databases were searched for literature published up to April 30, 2026. Compliance was defined as completed prompts divided by delivered prompts. Single-group proportions were pooled using logit transformation and random-effects models with the Hartung-Knapp-Sidik-Jonkman adjustment. Prediction intervals were calculated to describe the expected distribution of compliance in future comparable settings. Subgroup analyses, univariable meta-regressions, leave-one-out sensitivity analyses, and tests for small-study effects were performed. Risk of bias was assessed using the Joanna Briggs Institute Critical Appraisal Checklist for Studies Reporting Prevalence Data, methodological reporting quality was assessed using a modified Checklist for Reporting EMA Studies, and certainty of evidence was evaluated using the Grading of Recommendations Assessment, Development, and Evaluation approach.

**Results:**

Twenty-three studies involving 13,565 participants were included. The pooled compliance rate was 78.55% (95% CI 73.48%‐82.87%), with a prediction interval of 48.59%‐93.41%. Subgroup analyses identified no robust differences across study characteristics. Although study length showed a statistically significant subgroup test, the result was not stable after excluding singleton categories. Meta-regression analyses similarly found no significant linear associations for study length, prompts per day, items per prompt, or assessment window. Leave-one-out analyses showed that no single study drove the pooled estimate. Regarding the risk of bias, 2 studies were judged as low, while 21 were judged as moderate risk. Quality scores ranged from 6.5 to 9.0, and the certainty of evidence for the pooled compliance rate was rated as very low according to the Grading of Recommendations Assessment, Development, and Evaluation approach.

**Conclusions:**

Overall compliance with EMA among patients with cancer was moderate to high, suggesting that repeated real-world assessment may be feasible in oncology research settings. Nevertheless, the very high heterogeneity, wide prediction interval, and very low certainty of evidence indicate that compliance is context-dependent. The pooled estimate should therefore be interpreted as an approximate benchmark rather than a universal expected rate. Future oncology EMA studies should use standardized compliance denominators, report missing prompts transparently, and prospectively evaluate patient-centered design strategies that reduce burden while preserving data quality.

## Introduction

Cancer represents one of the most formidable global public health challenges, with its associated disease burden continuing to rise. Recent estimates indicate that the global cancer burden has surpassed 20 million new cases annually, a figure projected to surge to 35.3 million by 2050, driven by population growth and aging [1,2]. Patients with cancer experience a complex constellation of physical symptoms, psychological distress, and behavioral changes that exhibit pronounced temporal fluctuations and substantial interindividual variability [3-6]. Traditional cross-sectional surveys and retrospective designs inadequately capture this dynamic nature and are susceptible to recall bias [7], critically impeding nuanced understanding of patients’ real-world experiences and the development of personalized intervention strategies [8]. Approaches that capture symptoms, behaviors, and psychological states in real time within naturalistic contexts are therefore essential for identifying high-risk periods, tailoring individualized management, and designing effective interventions to improve treatment outcomes and quality of life.

Ecological momentary assessment (EMA) is a method that involves high-frequency, real-time sampling of individuals’ symptoms, behaviors, and emotions within their natural environments, generating data with high ecological validity [9,10]. Compared with traditional retrospective or single-timepoint assessments, EMA more accurately captures dynamic fluctuations in individual states [11]. Typically implemented through smartphones or wearable devices, EMA offers three major advantages: (1) reduced recall bias and enhanced data authenticity, (2) capture of temporal variability in symptoms, behaviors, and psychological states, and (3) collection of information in participants’ everyday settings, improving ecological validity [12-14]. EMA has been increasingly applied across diverse domains, including chronic disease management [15], mental health research [16], and oncology [17], offering new opportunities for personalized and precision-based interventions. For instance, Miller et al [15] used EMA to assess dyspnea in patients with chronic obstructive pulmonary disease, enabling real-time monitoring of symptom trajectories and exacerbation risks. Similarly, Henneghan et al [18] applied EMA among patients with breast cancer to capture symptoms and conduct mobile-based cognitive testing, providing continuous insights into cognitive changes over time.

Despite its advantages, the use and data quality of EMA studies depend heavily on participant compliance, the extent to which participants adhere to high-frequency, real-time reporting protocols [19], typically operationalized as the proportion of completed prompts [20]. Compliance in oncology is uniquely challenged by intensive assessment schedules, significant symptom burden, and fluctuating motivation [21,22]. Consequently, reported compliance rates among patients with cancer varied substantially, from 44.7% [23] to 99% [24]. A scoping review suggested that EMA is feasible in oncology but provided no quantitative synthesis of compliance or investigation of factors driving this heterogeneity [17]. This evidence gap stands in stark contrast to established compliance rates in other populations: 75.06% in substance use disorders [25], 86.41% in older adults [26], and 78.3% in children and adolescents [27]. Oncology populations present unique challenges that limit the generalizability of these findings. Specifically, patients with cancer experience fluctuating and multidimensional symptom clusters, including severe cancer-related fatigue [3], which can significantly hinder the executive function required for consistent EMA reporting. Furthermore, the acute toxicity and cyclic nature of anticancer therapies (eg, chemotherapy or immunotherapy infusions) create periods of extreme physical debilitation [6]. Combined with existential distress from a life-threatening diagnosis, these factors may uniquely compromise sustained motivation and capacity for intensive, real-time monitoring [6]. Therefore, a critical gap remains: the field lacks a rigorous quantitative synthesis providing an approximate benchmark for EMA compliance in cancer populations and identifying modifiable study design characteristics that optimize it. To address this, we conducted a systematic review and meta-analysis to provide evidence-based recommendations for future EMA implementation in oncology.

This systematic review had three objectives. First, to systematically characterize the key features of EMA studies in cancer populations. Second, to quantify overall compliance rates and examine how study design characteristics (eg, daily prompt frequency, study duration, and items per prompt) and procedural factors (eg, incentives and device type) influence compliance. Third, to discuss implications for optimizing EMA design and reporting in oncology research.

## Methods

### Protocol and Registration

The review protocol, including the search strategy, was prospectively registered in the International Prospective Register of Systematic Reviews (PROSPERO, CRD420251047109). This systematic review and meta-analysis were conducted in accordance with the PRISMA (Preferred Reporting Items for Systematic Reviews and Meta-Analyses) 2020 [28] and PRISMA-S (Preferred Reporting Items for Systematic Reviews and Meta-Analyses literature search extension) [29]. The completed PRISMA 2020 checklist and PRISMA-S checklist are provided in Checklists 1 and 2.

### Literature Search

We conducted a comprehensive literature search across multiple electronic databases, including Web of Science, PubMed, Embase, Cochrane Library, CINAHL, PsycINFO, CNKI, and Wanfang databases from inception to April 30, 2026. CINAHL and PsycINFO were searched simultaneously via the EBSCOhost platform, whereas the other databases were searched separately through their respective platforms.

The initial search strategies were independently developed by our research team based on a thorough review of relevant literature [13,17,19], and were further refined through intensive group discussions and consultations with information specialists. The search strategy used a combination of MeSH terms and free-text keywords related to (1) EMA (eg, “experience sampling,” “momentary assessment,” and “daily diary”) and (2) oncology (eg, “neoplasm,” “cancer,” and “tumor”). Additionally, the reference lists of relevant systematic reviews and meta-analyses were manually screened to identify further eligible studies. Furthermore, during the manuscript revision stage, we reran the exact search strategies across all databases to update the literature and ensure the inclusion of the most recent publications. The detailed search strategies for each database are provided in Multimedia Appendix 1.

No study registries, search filters, or additional sources (eg, gray literature, conference proceedings, and organizational websites) were searched or applied. The search strategy did not undergo formal peer review. Apart from database searching, manual screening of reference lists, and author contact for missing or unclear compliance data, no additional search methods were used.

### Inclusion and Exclusion Criteria

Studies were considered eligible for inclusion if they met the following criteria: (1) Population: Patients had a pathologically confirmed diagnosis of cancer. (2) Methodology: The study used EMA or the experience sampling method, including electronic daily diaries. (3) Outcome measures: clear quantitative documentation of EMA compliance metrics. (4) Study design: observational studies. (5) Sampling frequency: data were collected at least once per day. (6) Publication requirement: peer-reviewed articles in English or Chinese with full-text available.

Studies were excluded based on the following predefined criteria: (1) nonempirical studies: commentaries, editorials, conference abstracts, and studies lacking original data. (2) Intervention-embedded protocols: real-time assessments conducted as integral components of experimental interventions (eg, EMA used to deliver a treatment), as these may confound compliance attributable to assessment procedures alone. (3) Duplicate datasets: Secondary publications derived from identical EMA datasets (retained only the earliest publication per dataset). (4) Dyadic or multiparticipant studies: Studies that simultaneously monitored patients and their caregivers (eg, spouses or family members), as the primary focus of this meta-analysis was the independent compliance of oncology patients.

### Study Selection

Identified references were uploaded into Rayyan, a web-based review management tool for literature screening. All duplicates were removed in Rayyan. Two authors (MGY and DC) screened the titles and abstracts independently to identify potentially eligible studies. Then all full-text articles were assessed according to the inclusion and exclusion criteria by both reviewers. Disagreements were resolved through discussion and, when necessary, consultation with a senior reviewer (GC). Full-text studies that did not meet the inclusion criteria were excluded with reasons, and the study selection process was described using the PRISMA flow diagram.

### Data Extraction

Data from included studies were independently extracted using the collection form developed based on the adapted STROBE (Strengthening the Reporting of Observational Studies in Epidemiology) Checklist for Reporting EMA Studies (CREMAS) [30]: (1) Basic information included title, first author, year of publication, country, study purpose, study duration, and sample size. (2) Demographic characteristics included age, gender, cancer type, cancer stage, treatment, clinical status, etc (3) EMA-related features included EMA device type, application name, operating system, EMA data sampling scheme (time-based or event-based), duration of individual assessments, number of items (ie, number of items to be answered for each measurement), daily measurement frequency, assessment window (ie, the length of time that each measurement had a chance to be answered before it was considered unanswered), training for the EMA, incentive, participation rate, completed prompts (the number of prompts answered), delivered prompts (the number of prompts sent), attrition, and strategy to deal with unanswered prompts. The compliance rate was calculated as completed prompts divided by delivered prompts. For studies that reported only compliance rates without explicitly providing the number of answered prompts, we derived the answered prompts by multiplying the reported compliance rate by the total number of prompts (ie, scheduled study d and the planned daily assessments). For studies that did not report detailed data related to compliance, we attempted to contact their corresponding authors by email.

### Risk of Bias and Quality Assessment

#### Risk of Bias

Risk of bias was assessed using the Joanna Briggs Institute (JBI) Critical Appraisal Checklist for Studies Reporting Prevalence Data [31]. The checklist includes nine domains assessing the appropriateness of the sample frame, sampling method, sample size, description of study participants and setting, coverage of the analyzed sample, validity and reliability of outcome measurement, appropriateness of statistical analysis, and adequacy of response rate. Each item was rated as “yes,” “no,” “unclear,” or “not applicable.” Overall risk of bias was judged as low, moderate, or high based on the number and importance of domains rated as “no” or “unclear.” In this review, the “condition” in the JBI checklist was operationalized as EMA compliance, defined as completed prompts divided by delivered prompts.

#### Quality Assessment

The quality assessment of the included studies was completed independently by two authors (MGY and DC) using a modified version of the CREMAS, and disagreements were resolved through discussion. The checklist addresses the following nine dimensions: EMA technology, training program for EMA, study duration, prompting design, prompt frequency, attrition, latency, missing data, and limitations. Each dimension was scored using a 3-tier scoring system (0=not described, 0.5=partially described, 1=fully described) with a total score range of 0‐9. Higher scores indicate better methodological quality of the study.

#### GRADE Evaluation

The certainty of evidence for the primary outcome was assessed using the Grading of Recommendations Assessment, Development, and Evaluation (GRADE) approach. The GRADE assessment was conducted at the outcome level for the pooled EMA compliance rate. The following domains were considered: risk of bias, inconsistency, indirectness, imprecision, and publication bias. Certainty of evidence was rated as high, moderate, low, or very low. Because this review synthesized single-arm observational EMA studies rather than randomized intervention studies, the assessment focused on the certainty of evidence for the pooled compliance proportion.

#### Data Analysis

Meta-analyses were performed using R (version 4.5.1) via RStudio, primarily using the “meta” and “metafor” packages. Statistical significance was set at a 2-sided *P*<.05. Compliance was defined as the ratio of completed-to-delivered prompts. To stabilize variances and normalize the distribution, proportion data underwent logit transformation before synthesis and were subsequently back-transformed for clinical interpretation.

We used random-effects models for all analyses, incorporating the Hartung-Knapp-Sidik-Jonkman (HKSJ) adjustment to produce robust 95% CIs and minimize type I errors [32]. Between-study heterogeneity was assessed using Cochran Q, *I*^2^, and τ² statistics. Additionally, 95% prediction intervals were calculated to estimate the expected compliance range in future clinical settings [33].

To explore heterogeneity, prespecified subgroup analyses and exploratory univariable meta-regressions were conducted on logit-transformed scales, examining moderators such as study duration, prompt frequency, and assessment burden. The robustness of results was validated through leave-one-out sensitivity analyses. Finally, small-study effects and publication bias were evaluated via visual inspection of funnel plots and statistically confirmed using Egger and Begg tests.

#### Deviations From Protocol

Several deviations from the original study protocol were made during the implementation of this review to ensure methodological robustness. First, the search strategy was expanded by incorporating a broader range of keywords related to real-time digital monitoring and cancer-related symptom terminology to improve retrieval sensitivity. Second, the eligibility criteria were further refined to exclude studies where EMA was strictly embedded within therapeutic interventions or involved dyadic participant monitoring (eg, patient-caregiver pairs). These refinements were implemented to isolate the independent compliance behaviors of oncology patients and ensure the conceptual clarity of the pooled estimates.

## Results

### Study Selection

**Figure 1.**
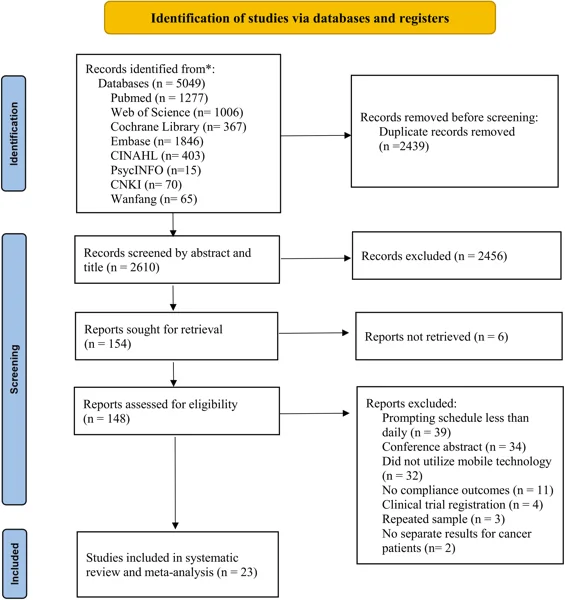
PRISMA (Preferred Reporting Items for Systematic Reviews and Meta-Analyses) 2020 flow diagram of the study selection process.

The database search identified 5049 records. After duplicate removal and title and abstract screening, full-text articles were assessed for eligibility, and 23 studies [18,21,34-54] were included in the final synthesis, with a total sample of 13,565 participants. Figure 1 presents the detailed process of study selection, Table 1 presents the characteristics of the included studies, and Table 2 presents the detailed information of the EMA design. We assessed potential overlap in study populations and confirmed that all included studies were based on independent samples, with no evidence of duplicate data.

**Table 1.** Characteristics of the included studies.

Study	Year	Country	Sample size, n	Age^a^, years	Female, n (%)	Cancer type	Clinical status	Attrition, n/N (%)
Geeraerts et al [34]	2026	Belgium	36	65.4 (10.3)	26 (72.2)	Advanced breast/lung cancer	Outpatient	4/40 (10)
Psihogios et al [35]	2021	United States	18	17.94 (2.31); range 15‐22	4 (22.2)	Leukemia	Outpatient	0/18 (0)
van Roozendaal et al [36]	2023	Netherlands	30	50.4 (9.7)	23 (76.7)	Mixed	Outpatient	7/37 (18.9)
Daniel et al [37]	2025	United States	407	48.73 (12.23)	367 (90.2)	Mixed cancer	Outpatient	19/426 (4.5)
Zwanenburg et al [38]	2025	Netherlands	61	58.26 (11.62)	33 (54.1)	Advanced lung cancer; melanoma	Outpatient	11/72 (15.3)
Panjer et al [39]	2026	Netherlands	33	67 (range 29‐76)	17 (52)	Mixed	Outpatient	3/33 (9.1)
Cobden et al [40]	2025	Australia	19	51.93 (9.31)	19 (100)	Breast cancer	Outpatient	0/19 (0)
King-Dowling et al [41]	2025	United States	20	18.9 (2.2)	9 (45)	Mixed childhood cancer survivors	Outpatient	2/22 (9.1)
Villegas et al [42]	2021	Spain	21	56.95 (10.53)	4 (19)	NR^b^	Outpatient	NR
Hacker and Ferrans [21]	2007	United States	20	48.7 (range 23‐64)	11 (55)	Hematologic malignancies	Inpatient	3/20 (15)
Ratcliff et al [43]	2014	United States	20	54.70 (10.29)	20 (100)	Breast cancer	Outpatient	1/21 (4.8)
Solk et al [44]	2019	United States	75	48.5 (10.3)	75 (100)	Breast cancer	Outpatient	12/75 (16)
Henneghan et al [18]	2025	United States	51	51.1 (11.1)	51 (100)	Metastatic breast cancer	Outpatient	1/52 (1.9)
Bates-Fraser et al [45]	2025	United States	40	63 (7)	40 (100)	Endometrial cancer	Outpatient	4/40 (10)
Shiyko et al [46]	2019	United States	59	66.1 (7.9)	36 (61)	Lung cancer	Outpatient	12/71 (16.9)
Badr et al [47]	2006	United States	56	Breast cancer: 56.7 (10.2); ovarian cancer: 58.3 (11.1)	56 (100)	Breast/ovarian cancer	Outpatient	NR
Stephenson et al [48]	2018	United States	53	49.38 (10.76); range 30‐73	53 (100)	Metastatic breast cancer	Outpatient	6/59 (10.2)
Hacker et al [49]	2017	United States	25	53.4 (11.5)	11 (44)	Hematologic malignancies	Outpatient	0/25 (0)
Pinto et al [50]	2021	United States	22	51.5 (8.4)	NR	Breast cancer	Outpatient	2/22 (9.1)
Heathcote et al [51]	2022	United States	29	17.6	14 (46.7)	Mixed childhood cancer survivors	Outpatient	1/30 (3.3)
Gustavell et al [52]	2019	Sweden	6	65 (range 57‐74)	3 (50)	Pancreatic/periampullary cancer	Outpatient	0/6 (0)
Cracchiolo et al [53]	2024	United States	12,433	57 (47‐65)	7874 (63.3)	Mixed	Outpatient	1619/12433 (13)
Komarzynski et al [54]	2019	France	31	61 (range 35‐91)	14 (45.2)	Mixed advanced cancer	Outpatient	1/31 (3.2)

a Age values are presented as mean (SD), median (IQR), or median/range as reported in the original studies.

b NR: not reported.

**Table 2.** Ecological momentary assessment characteristics. “Mixed” prompting scheme refers to studies using both time-based and event-based assessments.

Study	Items per prompt	Prompts per day	Latency (minutes)	Study length (days)	Prompting scheme	Training	Incentive	Device	Compliance
Geeraerts et al [34]	16	10	NR^a^	7	Time-based	NR	NR	Smartphone	77.6% (1676/2160)
Psihogios et al [35]	14	1	NR; 60-min reminder	28	Time-based	NR	Yes	SMS+MEMS^b^ TrackCap	88.9% (448/504)
van Roozendaal et al [36]	20	5	30	≥21	Time-based	Yes	NR	Smartphone	83.5% (2631/3150)
Daniel et al [37]	8	3	120	35	Time-based	NR	Yes	Smartphone	54.9% (23472/42735)
Zwanenburg et al [38]	NR	8	45	14	Time-based	Yes	Yes	Smartphone	89.1% (6085/6832)
Panjer et al [39]	22	3	45	42	Time-based	NR	Yes	Smartphone or web platform	67.0% (2533/3780)
Cobden et al [40]	NR	1	~5 min/session	30	Time-based	NR	Yes	Smartphone	82.1% (468/570)
King-Dowling et al [41]	NR	4	14.2; median 8.0	14	Time-based	NR	Yes	Smartphone+accelerometer	78.2% (862/1102)
Villegas et al [42]	8	≥2	NR	30	Mixed	Yes	NR	Smartphone	82.2% (1036/1260)
Hacker et al [21]	1	3	NR	6	Time-based	Yes	NR	Actigraphy watch	87.1% (290/333)
Ratcliff and Ferrans [43]	5/16	4	2‐4 min/session	21	Time-based	N/R	Yes	PDA^c^	57.3% (923/1612)
Solk et al [44]	7‐13	4	13.8 (4.6)	30	Time-based	Yes	Yes	SMS+accelerometer	86.0% (6502/7560)
Henneghan et al [18]	15	1	11.88 (1.60)	28	Time-based	Yes	Yes	Smartphone	94.0% (1342/1428)
Bates-Fraser et al [45]	NR	9	15	7	Time-based	Yes	Yes	Smartphone+accelerometer	69.0% (1565/2268)
Shiyko et al [46]	4	2	10	14	Time-based	Yes	NR	PDA	61.0% (1007/1652)
Badr et al [47]	18/19	4	2‐3 min/session	7/21	Time-based	Yes	NR	PDA	82.8% (2828/3416)
Stephenson et al [48]	2/3	6	2‐5 min/session	14	Time-based	Yes	Yes	PDA	70.2% (3127/4452)
Hacker et al [49]	1	5	NR	7	Time-based	Yes	NR	Actigraphy watch	79.1% (692/875)
Pinto et al [50]	22/23	5	NR	7	Time-based	Yes	Yes	Smartphone+accelerometer	78.6% (3027/3850)
Heathcote et al [51]	NR	3	2.55 (1.15)	11	Time-based	Yes	Yes	Smartphone	82.4% (789/957)
Gustavell et al [52]	12	1	NR	28	Time-based	Yes	NR	Smartphone	83.9% (141/168)
Cracchiolo et al [53]	14	1	1.7 (1.2‐2.5)	10	Time-based	Yes	NR	Computer or tablet or smartphone	58.8% (65274/110936)
Komarzynski et al [54]	19	1	NR	30	Time-based	Yes	NR	Computer+actigraph	74.8% (696/930)

a NR: not reported.

b MEMS: medication event monitoring system.

c PDA: personal digital assistant.

### Participant Characteristics

Participants were predominantly female (64.7%, 8760/13,543 among studies reporting sex). The included studies were conducted across 7 countries, with the United States contributing the majority (n=15, 65.2%) [18,21,35,37,41,43-51,53]. Sample sizes varied substantially, ranging from 6 participants to 12,433 participants. Most studies were conducted in outpatient settings (n=22, 95.7%) [18,34-54]. Additional characteristics of the study populations are summarized in Table 1. (More comprehensive information can be found in Multimedia Appendix 2)

### Study Characteristics

#### Study Length

Among the 23 included studies [18,21,34-54], the assessment period ranged from 6 to 42 days. Overall, 11 studies [21,34,38,41,45,46,48-51,53] lasted 7‐14 days, and 11 studies [18,35-37,39,40,42-44,52,54] lasted more than 14 days.

#### Sampling Scheme

Sampling schemes were classified as time-based, event-based, or mixed. Most included studies used time-based sampling [18,21,34-41,43-54] (22/23, 95.7%), while 1 study [42] used a mixed time-based and event-based design.

#### Sampling Frequency

Sampling frequency ranged from 1 to 10 prompts per day. Low-frequency sampling (1‐3 prompts per d) was used in 12 studies [18,21,35,37,39,40,42,46,51-54], moderate-frequency sampling (4‐5 prompts per d) in 7 studies [36,41,43,44,47,49,50], and high-frequency sampling (≥6 prompts per d) in 4 studies [34,38,45,48].

#### Number of Items

The number of items per assessment varied considerably. Eight studies [21,37,42-44,46,48,49] used fewer than 10 items per assessment, 8 studies [18,34-36,43,44,47,54] used 10‐20 items, 2 studies [39,50] used more than 20 items, and 5 studies [38,40,41,45,51] did not report this information clearly.

#### Sampling Devices

With respect to device usage, smartphones were the most widely used platform (15/23, 65.2%). Four studies [43,46-48] used personal digital assistants and 4 studies [21,35,44,49] used other platforms, such as patient portals, computers, or dedicated monitoring devices.

#### Training

Reporting of implementation details was incomplete. Sixteen studies [18,21,36,38,42,44-54] explicitly reported participant training, whereas 7 studies [34,35,37,39-41,43] did not report this information.

#### Incentive

Thirteen studies [18,35,37-41,43-45,48,50,51] reported providing incentives or compensation to participants, whereas 10 studies [21,34,36,42,46,47,49,52-54] did not report this aspect.

#### Assessment Window

With regard to the assessment window, 11 studies [21,34,35,40,42,43,47,49,50,52,54] did not report the allowable time frame for completing prompts. Among studies that did report this information, 6 studies [36,38,39,45,46,48] used windows shorter than 1 hour, 4 studies [37,41,44,51] used windows of 1‐3 hours, and 2 studies [18,53] used windows longer than 3 hours (Table 2).

### Meta-Analysis

#### Compliance Rate

**Figure 2.**
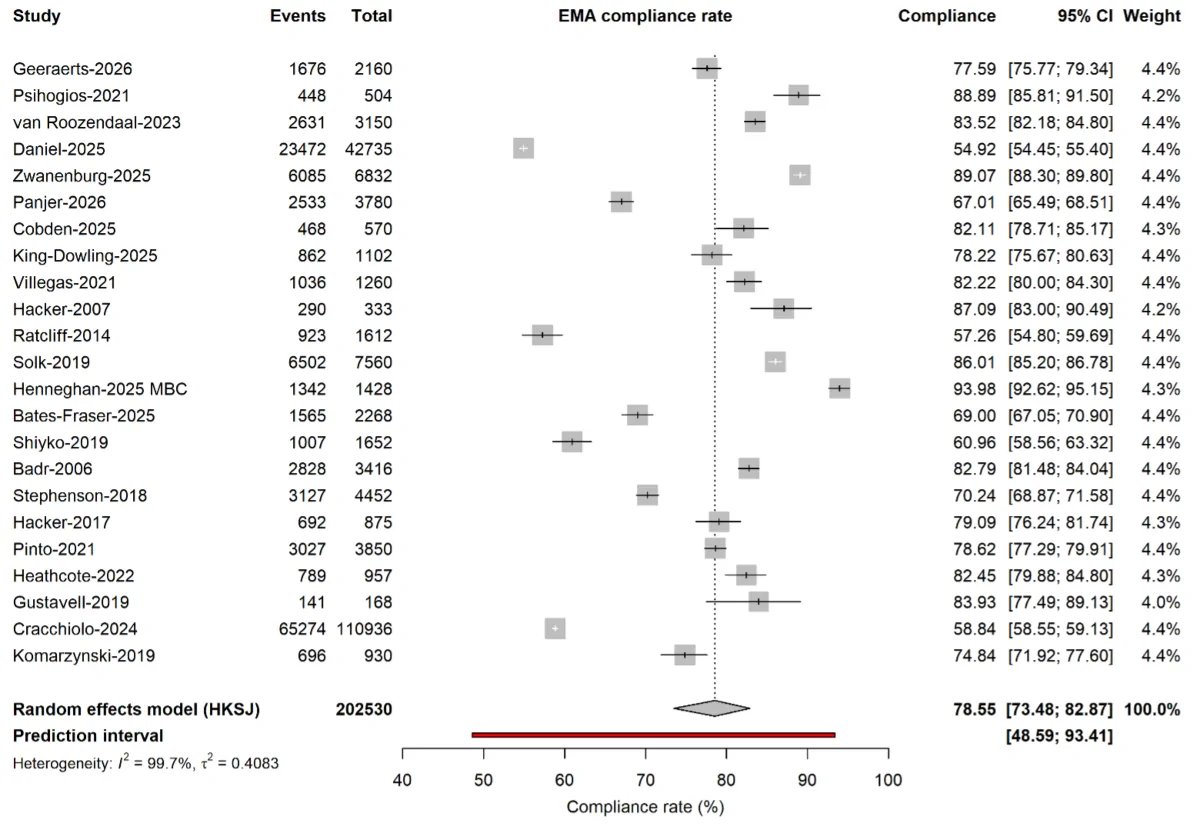
Forest plot of ecological momentary assessment compliance rates among patients with cancer [18,21,34-54]. EMA: ecological momentary assessment.

In the meta-analysis, a total of 23 studies [18,21,34-54] were included, yielding a pooled compliance rate of 78.55% (95% CI 73.48%‐82.87%) among patients with cancer. The prediction interval ranged from 48.59% to 93.41%. Substantial heterogeneity was observed (*I*²=99.7%, τ²=0.4083, Q=8043.84; *P*<.001). Detailed results are presented in Figure 2.

#### Subgroup Analyses

**Figure 3.**
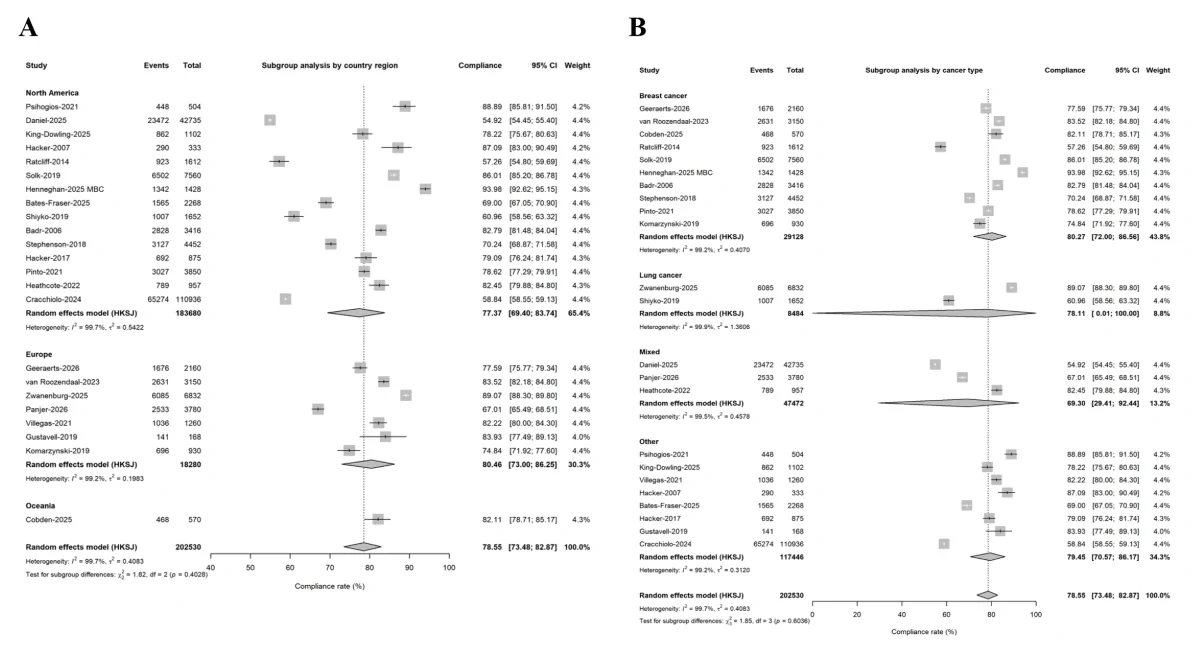
Subgroup analyses of compliance rates for country region (A) and cancer type (B) [18,21,34-54].

**Figure 4.**
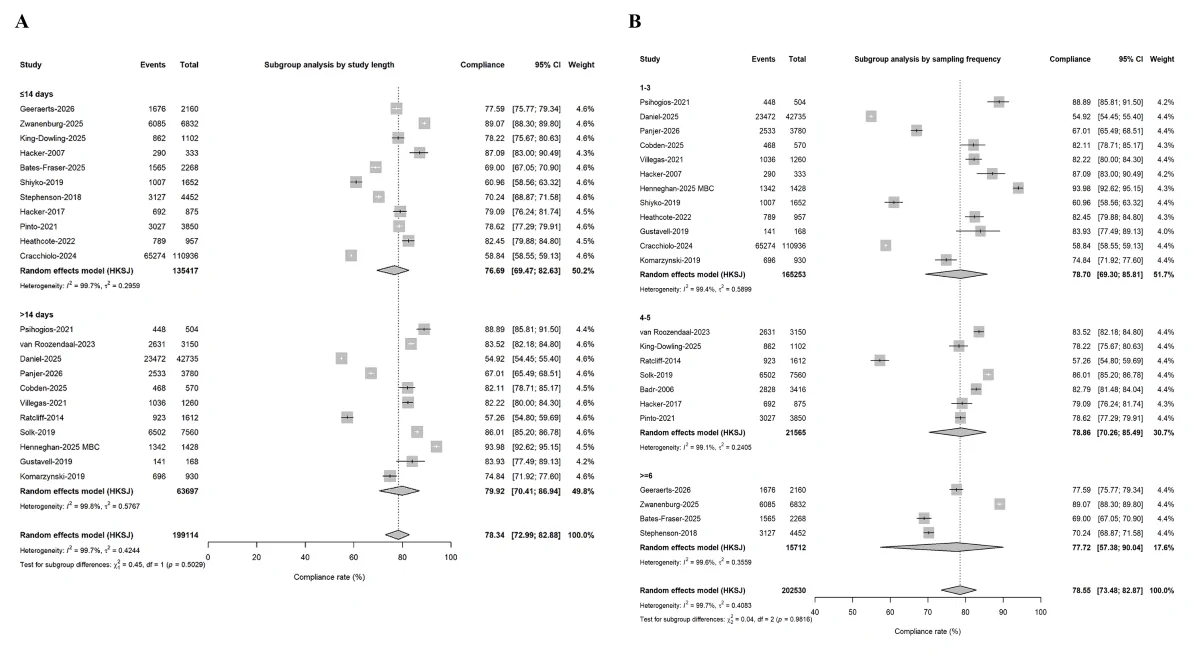
Subgroup analyses of compliance rates for study length (A) and sampling frequency (B) [18,21,34-54].

**Figure 5.**
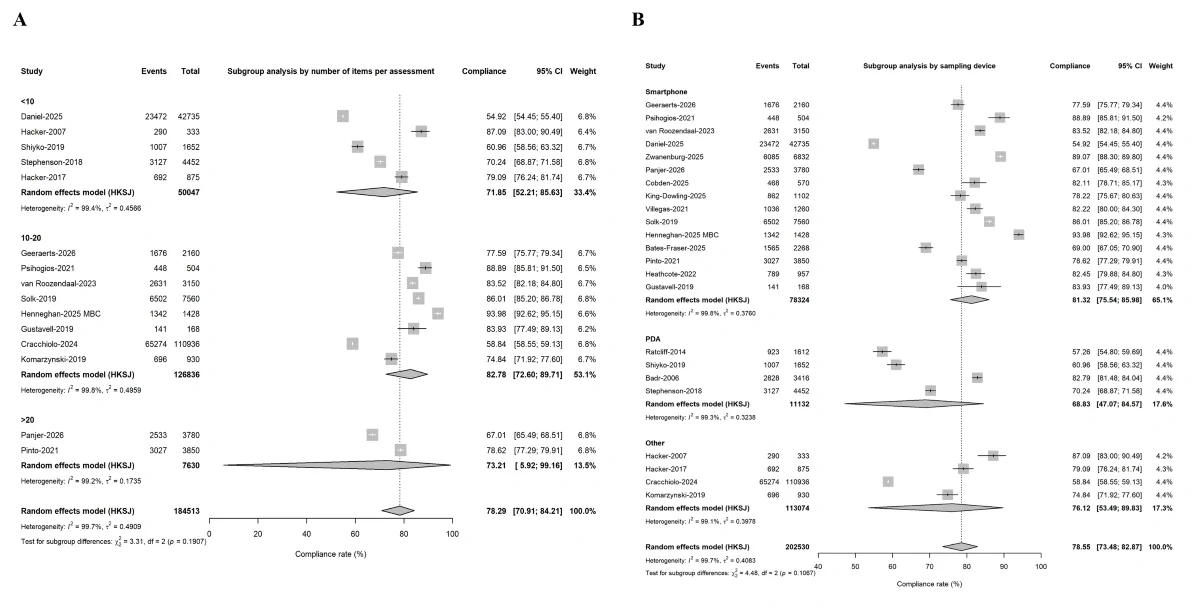
Subgroup analyses of compliance rates for number of items (A) and sampling devices (B) [18,21,34-54].

**Figure 6.**
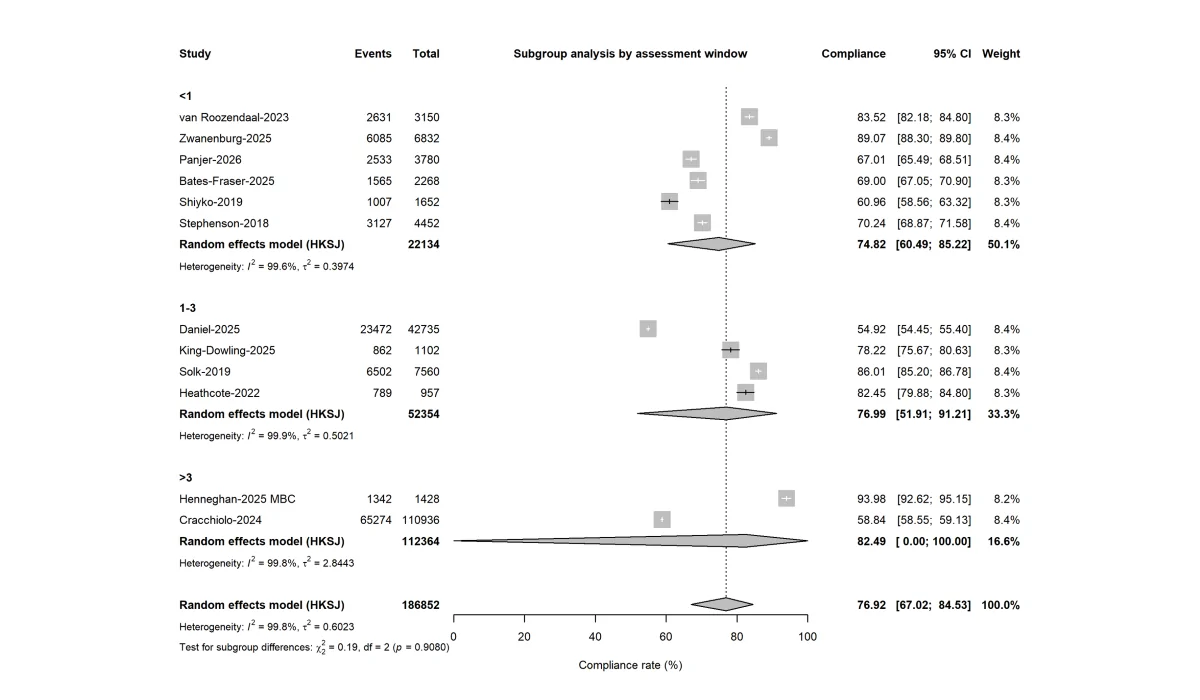
Subgroup analyses of compliance rates for assessment window [18,36-39,41,44-46,48,51,53].

We conducted subgroup analyses across various EMA characteristics to further examine the impact of common study design features or procedural factors on compliance. Detailed results are provided in Table 3 and Figures 3–6. No robust subgroup differences were observed by country region, cancer type, study length, sampling frequency, items per assessment, sampling devices, and assessment window. Some features were not included in subgroup analyses because their category distributions were highly imbalanced.

**Table 3.** Subgroup analyses of compliance rates for categorical moderator variables. Subgroup analyses were exploratory and used a logit-transformed random-effects model with Hartung-Knapp adjustment and the Sidik-Jonkman estimator.

Variable	Studies, n (%)	Compliance rate (95% CI) (%)	Heterogeneity test–*I^2^*	Test for subgroup differences	
				Q value (*df*)	*P* value
Country region			99.7%	1.82 (2)	.40
North America	15 (65.2)	77.37 (69.40‐83.74)	99.7%		
Europe	7 (30.4)	80.46 (73.00‐86.25)	99.2%		
Oceania	1 (4.3)	82.11 (78.74‐85.04)	—^a^		
Cancer type			99.7%	1.85 (3)	.60
Breast cancer	10 (43.5)	80.27 (72.00‐86.56)	99.2%		
Lung cancer	2 (8.7)	78.11 (0.01‐100.00)	99.9%		
Mixed	3 (13)	69.30 (29.41‐92.44)	99.5%		
Other	8 (34.8)	79.45 (70.57‐86.17)	99.2%		
Study length (days)			99.7%	0.45 (1)	.50
≤14	11 (50)	76.69 (69.47‐82.63)	99.7%		
>14	11 (50)	79.92 (70.41‐86.94)	99.8%		
Sampling frequency (prompts/day)			99.7%	0.04 (2)	.98
1‐3	12 (52.2)	78.70 (69.30‐85.81)	99.4%		
4‐5	7 (30.4)	78.86 (70.26‐85.49)	99.1%		
≥6	4 (17.4)	77.72 (57.38‐90.04)	99.6%		
Items per assessment			99.7%	3.31 (2)	.19
<10	5 (33.3)	71.85 (52.21‐85.63)	99.4%		
10‐20	8 (53.3)	82.78 (72.60‐89.71)	99.8%		
>20	2 (13.3)	73.21 (5.92‐99.16)	99.2%		
Sampling devices			99.7%	4.48 (2)	.11
Smartphone	15 (65.2)	81.32 (75.54‐85.98)	99.8%		
PDA^b^	4 (17.4)	68.83 (47.07‐84.57)	99.3%		
Other	4 (17.4)	76.12 (53.49‐89.83)	99.1%		
Assessment window (hours)			99.8%	0.19 (2)	.91
<1	6 (50)	74.82 (60.49‐85.22)	99.6%		
1‐3	4 (33.3)	76.99 (51.91‐91.21)	99.9%		
>3	2 (16.7)	82.49 (0.00‐100.00)	99.8%		

a Not available.

b PDA: personal digital assistant.

### Risk of Bias and Quality Assessment

#### Risk of Bias

2 studies [37,53] were judged as low risk of bias, and 21 studies [18,21,34-36,38-52,54] were judged as moderate risk of bias. The most common sources of concern were unclear sampling procedures and inadequate or unjustified sample size, which were common in pilot or feasibility EMA studies (Table 4).

**Table 4.** Risk of bias assessment using the Joanna Briggs Institute checklist for the included studies.

Study	Q1^a^	Q2^b^	Q3^c^	Q4^d^	Q5^e^	Q6^f^	Q7^g^	Q8^h^	Q9^i^	Overall risk
Geeraerts et al [34]	Y^j^	U^k^	N^l^	Y	Y	Y	Y	Y	Y	Moderate
Psihogios et al [35]	Y	U	N	Y	Y	Y	Y	Y	Y	Moderate
van Roozendaal et al [36]	Y	U	N	Y	Y	Y	Y	Y	Y	Moderate
Daniel et al [37]	Y	U	Y	Y	Y	Y	Y	Y	Y	Low
Zwanenburg et al [38]	Y	U	N	Y	Y	Y	Y	Y	Y	Moderate
Panjer et al [39]	Y	U	N	Y	Y	Y	Y	Y	Y	Moderate
Cobden et al [40]	Y	U	N	Y	Y	Y	Y	Y	Y	Moderate
King-Dowling et al [41]	Y	U	N	Y	Y	Y	Y	Y	Y	Moderate
Villegas et al [42]	U	U	N	U	U	Y	Y	Y	U	Moderate
Hacker and Ferrans [21]	Y	U	N	Y	Y	Y	Y	Y	Y	Moderate
Ratcliff et al [43]	Y	U	N	Y	Y	Y	Y	Y	Y	Moderate
Solk et al [44]	Y	U	N	Y	Y	Y	Y	Y	Y	Moderate
Henneghan et al [18]	Y	U	N	Y	Y	Y	Y	Y	Y	Moderate
Bates-Fraser et al [45]	Y	U	N	Y	Y	Y	Y	Y	Y	Moderate
Shiyko et al [46]	Y	U	N	Y	Y	Y	Y	Y	Y	Moderate
Badr et al [47]	Y	U	N	Y	U	Y	Y	Y	U	Moderate
Stephenson et al [48]	Y	U	N	Y	Y	Y	Y	Y	Y	Moderate
Hacker et al [49]	Y	U	N	Y	Y	Y	Y	Y	Y	Moderate
Pinto et al [50]	Y	U	N	Y	Y	Y	Y	Y	Y	Moderate
Heathcote et al [51]	Y	U	N	Y	Y	Y	Y	Y	Y	Moderate
Gustavell et al [52]	Y	U	N	Y	Y	Y	Y	Y	Y	Moderate
Cracchiolo et al [53]	Y	Y	Y	Y	Y	Y	Y	Y	Y	Low
Komarzynski et al [54]	Y	U	N	Y	Y	Y	Y	Y	Y	Moderate

a Q1: Was the sample frame appropriate to address the target population?

b Q2: Were study participants sampled in an appropriate way?

c Q3: Was the sample size adequate?

d Q4: Were the study subjects and setting described in detail?

e Q5: Was the data analysis conducted with sufficient coverage of the identified sample?

f Q6: Were valid methods used for the identification of EMA compliance?

g Q7: Was EMA compliance measured in a standard, reliable way for all participants?

h Q8: Was there appropriate statistical analysis?

i Q9: Was the response rate adequate, and if not, was the low response rate managed appropriately?

j Y: yes.

k U: unclear.

l N: no.

#### Quality Assessment

The methodological quality scores of the included studies ranged from 6.5 to 9.0, indicating generally moderate to high reporting quality. Most studies clearly described EMA technology, study duration, prompting design, prompt frequency, and attrition. However, several studies provided limited information on EMA training, response latency, or missing data handling, which were the main sources of lower scores (Table 5).

**Table 5.** Quality scores of the included studies.

Study	EMA^a^ technology	EMA training	Study duration	Prompting scheme	Frequency	Attrition	Latency	Missing data	Limitations of EMA	Total score
Geeraerts et al [34]	1^b^	0^c^	1	1	1	1	0	0.5^d^	1	6.5
Psihogios et al [35]	1	0	1	1	1	1	0.5	1	1	7.5
van Roozendaal et al [36]	1	1	1	1	1	1	1	1	1	9
Daniel et al [37]	1	0	1	1	1	1	1	1	1	8
Zwanenburg et al [38]	1	1	1	1	1	1	1	1	1	9
Panjer et al [39]	1	0	1	1	1	1	1	1	1	8
Cobden et al [40]	1	0	1	1	1	1	0.5	1	1	7.5
King-Dowling et al [41]	1	0.5	1	1	1	1	1	1	1	8.5
Villegas et al [42]	1	1	1	1	1	0.5	0	0.5	1	7
Hacker and Ferrans [21]	1	1	1	1	1	1	0	1	0.5	7.5
Ratcliff et al [43]	1	0.5	1	1	1	1	1	1	1	8.5
Solk et al [44]	1	1	1	1	1	1	1	1	1	9
Henneghan et al [18]	1	1	1	1	1	1	1	1	1	9
Bates-Fraser et al [45]	1	1	1	1	1	1	1	1	1	9
Shiyko et al [46]	1	1	1	1	1	1	1	1	0.5	8.5
Badr et al [47]	1	1	1	1	1	1	0.5	0.5	0.5	7.5
Stephenson et al [48]	1	1	1	1	1	1	1	1	0.5	8.5
Hacker et al [49]	1	1	1	1	1	1	0	1	1	8
Pinto et al [50]	1	1	1	1	1	1	0	1	1	8
Heathcote et al [51]	1	1	1	1	1	1	1	1	1	9
Gustavell et al [52]	1	1	1	1	1	1	0	0.5	1	7.5
Cracchiolo et al [53]	1	1	1	1	1	1	1	0.5	1	8.5
Komarzynski et al [54]	1	1	1	1	1	1	0	1	1	8

a EMA: ecological momentary assessment.

b Yes (fully described)=1.

c No (not described)=0.

d Somewhat (partially described)=0.5.

#### Certainty of Evidence Assessment

The certainty of this evidence was very low, downgraded for serious risk of bias (due to lack of randomization and potential selection bias), very serious inconsistency (substantial heterogeneity across studies), and serious indirectness (differences in EMA protocols, compliance definitions, and patient populations). No further downgrading was required for imprecision (the CI was reasonably narrow; Table 6).

**Table 6.** Grading of Recommendations Assessment, Development and Evaluation evidence profile for ecological momentary assessment compliance rate. Compliance was defined as the number of completed prompts divided by the total number of delivered or scheduled prompts. The certainty of evidence was assessed at the outcome level for the pooled ecological momentary assessment compliance rate.

Studies, n	Certainty assessment						Effect			Certainty	Importance
	Study design	Risk of bias^a^	Inconsistency^b^	Indirectness^c^	Imprecision^d^	Other considerations	Events, n	Individuals, n	Rate (95% CI)		
EMA^e^ Compliance rate											
23	Nonrandomized studies	Serious	Very serious	Serious	Not serious	none	NA^f^	13,565	Event rate 78.55% (73.48‐82.87)	Very low ⊕◯◯◯	8-critical

a Risk of bias: Downgraded for serious risk of bias because most included studies were single-arm observational, feasibility, or diary-based studies, and several studies incompletely reported key EMA implementation details, such as missing data handling, assessment windows, training procedures, or compliance calculation methods.

b Inconsistency: Downgraded for very serious inconsistency because heterogeneity was extremely high (I²=99.7%, τ²=0.4083), and the prediction interval was wide, suggesting substantial variability in compliance across future comparable real-world settings.

c Indirectness: Downgraded for serious indirectness because included studies varied substantially in cancer type, clinical status, EMA/ePRO design, device type, sampling frequency, assessment window, study duration, and compliance denominator definitions

d Imprecision: Not downgraded for imprecision because the 95% confidence interval around the pooled estimate was relatively narrow. The broad prediction interval was considered under inconsistency rather than imprecision.

e EMA: ecological momentary assessment

f NA: not applicable

#### Meta-Regression Analyses

**Figure 7.**
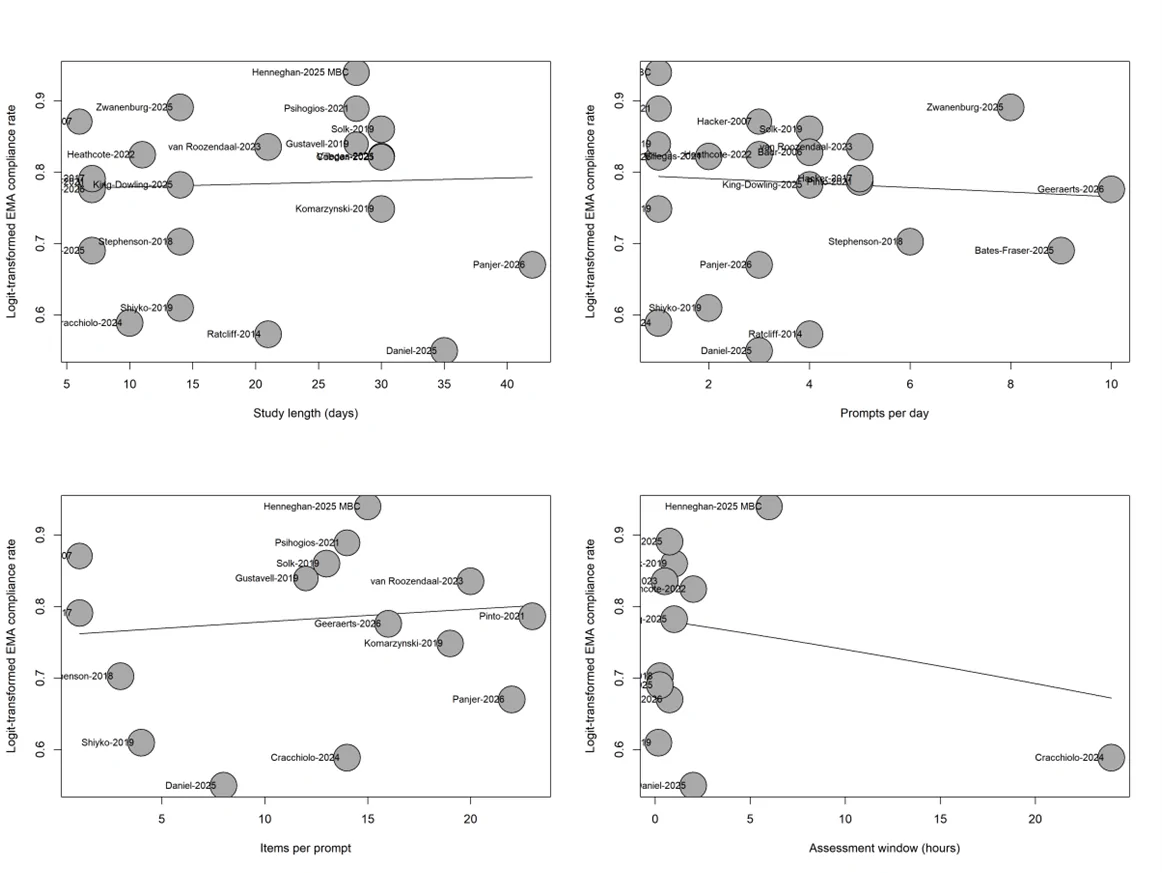
Meta-regression analyses of continuous ecological momentary assessment design characteristics [18,21,34-54]. EMA: ecological momentary assessment.

Univariable meta-regression analyses were conducted to examine whether continuous EMA design characteristics were associated with compliance rates. No significant linear associations were observed for study length (k=22; *β*=0.0025; *P*=.85), prompts per day (k=23; *β*=−0.0184; *P*=.74), number of items per prompt (k=15; *β*=0.0105; *P*=.70), or assessment window in hours (k=12; *β*=−0.0234; *P*=.53). Residual heterogeneity remained substantial across all models, indicating that these continuous design characteristics did not explain the high between-study variability in compliance rates (Table 7 and Figure 7).

**Table 7.** Univariable meta-regression of continuous ecological momentary assessment design characteristics. β coefficients are presented on the logit-transformed compliance proportion scale. Each model was fitted separately as a univariable random-effects meta-regression. Studies with unavailable moderator data were excluded from the corresponding model.

Moderator	Studies included, k	Range	*β* coefficient	*P* value
Study length, days	22	6‐42	0.0025	.85
Prompts per day	23	1‐10	−0.0184	.74
Items per prompt	15	1‐23	0.0105	.70
Assessment window, hours	12	0.17‐24	−0.0234	.53

#### Sensitivity Analysis

**Figure 8.**
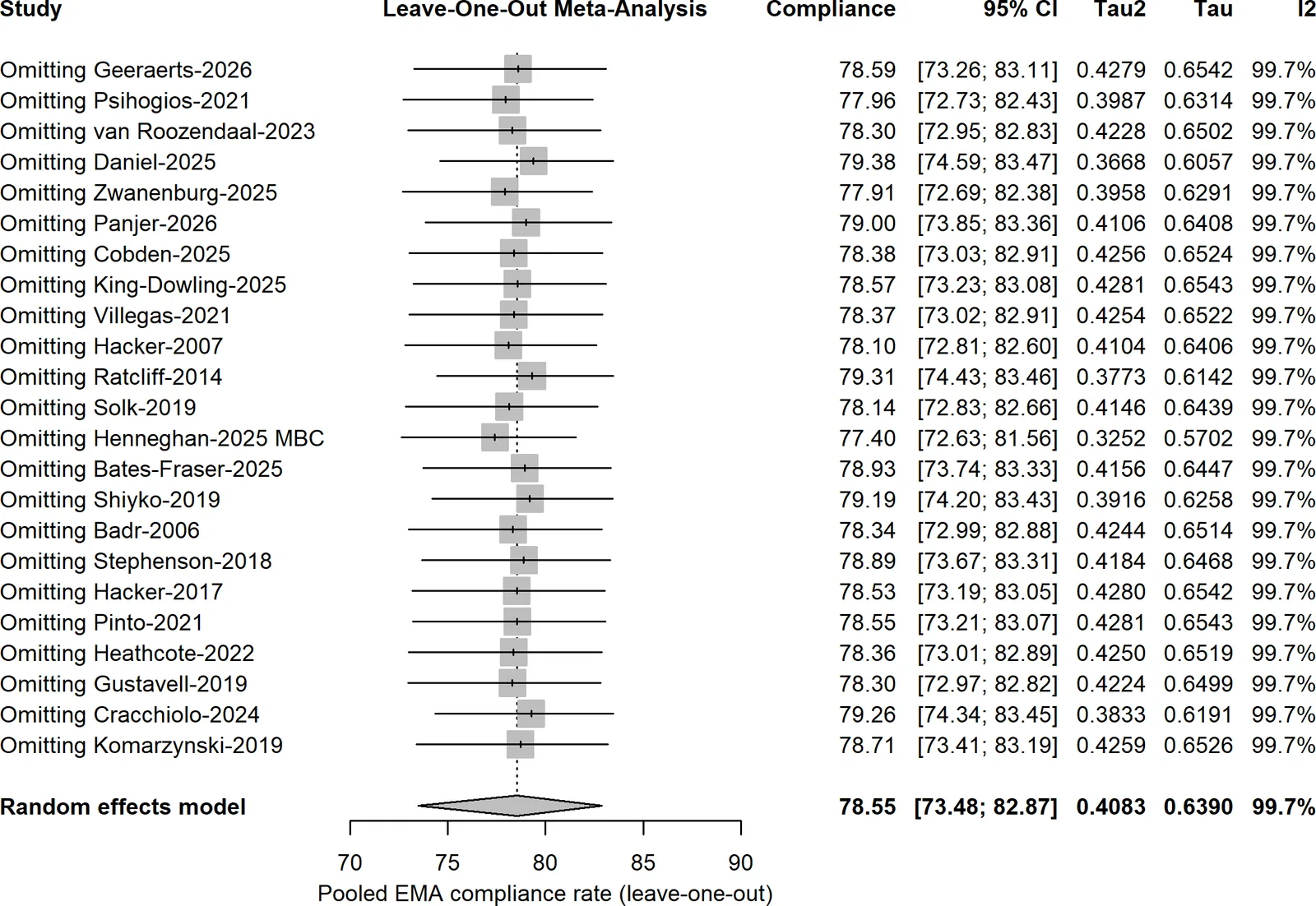
Leave-one-out sensitivity analysis of the pooled ecological momentary assessment compliance rate [18,21,34-54].

Sensitivity analysis was performed using leave-one-out meta-analysis to assess the robustness of the pooled estimate. After excluding each study sequentially, the pooled EMA compliance rates ranged from 77.40% to 79.38%, which was highly consistent with the overall pooled estimate of 78.55% (95% CI 73.48%‐82.87%). The heterogeneity remained consistently high across all iterations (*I*²=99.7%), and the between-study variance showed only modest variation (τ²=0.3252‐0.4281). These findings indicate that no single study exerted an undue influence on the overall pooled estimate, although substantial between-study heterogeneity persisted (Figure 8).

#### Reporting Biases and Small-Study Effects

**Figure 9.**
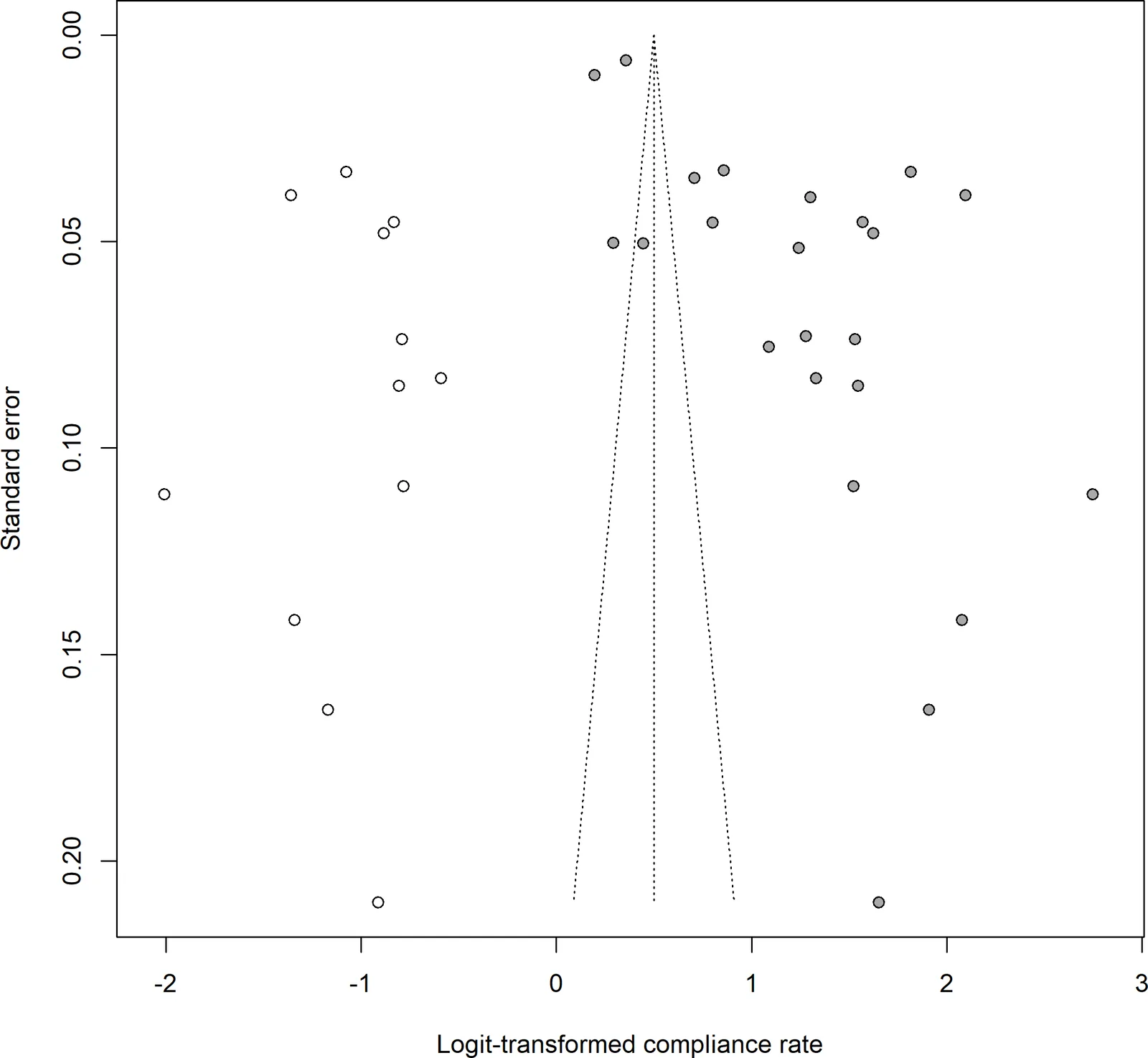
Funnel plot for small-study effects in ecological momentary assessment compliance estimates.

Potential reporting biases and small-study effects were assessed using funnel plots, Egger linear regression test, and Begg rank correlation test. Visual inspection of the funnel plot showed an uneven distribution of studies around the pooled logit-transformed estimate, with several studies with larger standard errors scattered outside the pseudo 95% confidence limits, suggesting possible funnel-plot asymmetry. Begg test indicated statistically significant small-study effects (*P*=.02), whereas Egger test was not statistically significant (*P*=.11). Therefore, the evidence for small-study effects was suggestive but not conclusive. Given the substantial between-study heterogeneity and the inconsistent findings between the two statistical tests, the funnel-plot asymmetry should be interpreted cautiously, and potential small-study effects or reporting bias cannot be ruled out (Figure 9).

## Discussion

### Principal Findings

To our knowledge, this is the first meta-analysis to quantitatively synthesize EMA compliance among oncology populations, directly addressing our three primary research objectives. In alignment with our first aim, we systematically characterized EMA deployment strategies across 23 studies [18,21,34-54]. Regarding our second objective, the pooled analysis revealed a generally favorable overall compliance within the moderate to high range [19]. However, contrary to our expectations, subgroup and meta-regression analyses indicated that no robust study-level design characteristics or procedural factors consistently explained compliance variability. Consequently, the wide prediction interval suggests that compliance remains highly context-dependent [55]. Finally, reflecting our third objective, while EMA is a feasible methodology for real-world symptom tracking in cancer care, this interpretation must remain cautious in light of substantial heterogeneity, risk of bias, and the low certainty of the current evidence.

The EMA compliance observed in this review was broadly comparable to existing EMA benchmarks and meta-analyses. Stone and Shiffman recommended an 80% compliance threshold as a useful methodological benchmark for EMA studies [56]. Our findings align closely with this threshold, as well as with previous meta-analyses reporting pooled compliance rates of approximately 75% in individuals with substance use disorders [25], 86% in older adults [26], and 78% in children and adolescents [27]. Specifically, the compliance among patients with cancer was slightly lower than the rates reported for older adults, but exceeded those found in individuals with substance use disorders and was comparable to the levels seen in pediatric and adolescent populations. The disparity may stem from the relatively regular daily routines of older adults and the lower EMA prompting frequency typically used in studies involving this demographic [57], which together facilitate sustained participation. In contrast, patients with cancer often contend with severe symptom burdens and treatment-induced discomfort [42,43,48], which may hinder their ability to complete all assessments promptly, resulting in slightly lower compliance relative to older adults. However, compared with individuals with substance use disorders or youth cohorts, patients with cancer tend to exhibit stronger health management motivation and well-established compliance behaviors shaped through rigorous medical follow-ups [58,59]. Furthermore, one possible explanation is that participants who enroll in EMA studies may be more motivated or more comfortable with health-related monitoring, although this cannot be confirmed using aggregate study-level data.

The notable gap between the high EMA compliance observed in this study and the typically lower rates of clinical treatment compliance (20%‐60%) [60] highlights unique behavioral dynamics. Crucially, the pooled compliance estimate should not be equated with clinical treatment compliance. EMA completion is a short-term research behavior involving repeated self-reported prompts [9,10], whereas treatment compliance is shaped by broader clinical, behavioral, and health-system factors [60]. This divergence likely stem from the low-burden nature of EMA tasks [12-14], the shorter durations [12-14], and a potential “Hawthorne effect” where frequent digital prompts foster sustained engagement [61]. While these findings suggest patients with cancer are capable of consistent real-time monitoring, selection bias must be acknowledged, as study participants may represent a more motivated subgroup with higher baseline compliance [62]. Nonetheless, EMA remains a high-fidelity tool for capturing the symptomatic triggers, such as acute pain or fatigue, that often precipitate clinical noncompliance [12-14].

Focusing solely on comparative averages may overlook the profound variability observed across oncology studies, as evidenced by the wide 95% prediction interval of 48.59%‐93.41%. This wide prediction interval reflects substantial between-study variation and highlights the context-dependent nature of EMA compliance in oncology populations. While such variability presents statistical challenges, the pooled estimate remains valuable as a benchmark when interpreted alongside the prediction interval [33], signaling that researchers should set context-specific targets rather than expecting uniform rates. Furthermore, the absence of significant predictors in our meta-regression suggests that compliance is likely governed by complex, nonlinear interactions among disease severity, treatment toxicity, and digital literacy, rather than simple design metrics like prompt frequency alone [19].

Subgroup analyses revealed that EMA compliance remained remarkably consistent across diverse study characteristics. Specifically, no significant differences were identified by country region, cancer type, or technical configurations such as device type and assessment windows. However, these null findings should be interpreted cautiously, as many subgroup comparisons were underpowered due to limited study counts within specific strata [63]. Similarly, factors such as study length, sampling frequency, and items per assessment did not significantly alter compliance rates. These findings may imply that oncology patients’ engagement with EMA may be driven more by intrinsic factors, such as health motivation and perceived utility, than by extrinsic protocol adjustments [64,65]. Consequently, researchers may have considerable flexibility in designing EMA protocols without necessarily compromising participant compliance, provided the clinical burden remains manageable.

Overall, these findings highlight that EMA is a feasible, scalable methodology for oncology populations, supporting its integration into personalized health management [66]. Future studies should focus on developing standardized reporting and compliance metrics to enhance cross-study comparability and reproducibility. Additionally, given that traditional design features did not significantly drive compliance in our analysis, future research should investigate more complex, nonlinear predictors of engagement, such as digital literacy and real-time symptomatic burden, through mixed-methods approaches. Finally, exploring the long-term sustainability of EMA in clinical practice is essential to maintain high-fidelity data collection throughout the cancer survivorship continuum without imposing excessive patient burden.

### Limitations

This study has several limitations. First, there was substantial heterogeneity among the included studies, and the available study-level variables did not fully explain this heterogeneity. Some subgroup analyses were constrained by small sample sizes, warranting cautious interpretation of these results. Second, many studies provided incomplete reporting of implementation details, such as participant training, incentive strategies, assessment windows, response latency, missing data handling, or the exact denominator used to calculate compliance. This lack of methodological transparency directly increases the potential risk of bias, particularly regarding selection and reporting biases. Third, this review included only published studies in English and Chinese, potentially introducing reporting biases and small-study effects. Finally, the exclusion of studies due to missing or nonextractable prompt-level compliance data may introduce a degree of reporting bias, as studies with lower compliance might be less likely to report these metrics. Consequently, these compounded factors, including severe inconsistency, potential reporting bias, and the observational nature of the primary literature, resulted in a very low to low certainty of evidence within the GRADE framework. Therefore, our pooled compliance estimate should be interpreted as an approximate, context-dependent benchmark rather than a universal standard.

### Conclusions

This study suggests that patients with cancer can achieve moderate-to-high compliance with EMA protocols. However, the wide prediction interval, substantial heterogeneity, possible reporting bias, and very low certainty of evidence indicate that compliance is highly context-dependent. Consequently, the pooled estimate should therefore be interpreted as an approximate benchmark for planning oncology EMA studies rather than as a universal expected rate. For future research, maximizing feasibility requires careful, transparent, and patient-centered implementation. Researchers should consistently report compliance denominators, distinguish delivered from prompts, and clarify response windows alongside missing data handling. Furthermore, comprehensive reporting on training protocols, incentives, and attrition patterns is essential. Prospective studies must evaluate how varying combinations of design features impact compliance across distinct clinical cohorts and cancer stages. In terms of translation, EMA holds substantial value for capturing real-time symptoms, behaviors, and psychological states in patients’ daily lives. However, before widespread adoption in routine oncology care, identifying sustainable designs that preserve data quality without exacerbating patient burden remains a critical priority. Standardized reporting, enhanced methodological transparency, and a deeper understanding of patient-level compliance determinants are pivotal to transitioning EMA from a promising research tool to a reliable clinical monitoring strategy.

## Supplementary material

10.2196/87506 Multimedia Appendix 1 Search strategies.

10.2196/87506 Multimedia Appendix 2 Characteristics of included studies.

10.2196/87506 Checklist 1 PRISMA checklist.

10.2196/87506 Checklist 2 PRISMA-S checklist.

## Abbreviations

CREMAS: Checklist for Reporting EMA Studies
EMA: ecological momentary assessment
GRADE: Grading of Recommendations Assessment, Development and Evaluation
HKSJ: Hartung-Knapp-Sidik-Jonkman
JBI: Joanna Briggs Institute
PRISMA: Preferred Reporting Items for Systematic Reviews and Meta-Analyses
PRISMA-S: Preferred Reporting Items for Systematic Reviews and Meta-Analyses literature search extension
PROSPERO: International Prospective Register of Systematic Reviews
STROBE: Strengthening the Reporting of Observational Studies in Epidemiology
