# Supplementary material for: Compliance With Ecological Momentary Assessment Among Patients With Cancer: Systematic Review and Meta-Analysis
Source: J Med Internet Res. 2026 Sep 8;28:e87506. doi: 10.2196/87506 (PMC13552840; doi:10.2196/87506)
Supplement: Supplementary file 3 [file jmir-v28-e87506-s001.docx]

# Search strategies

## Web of Science

**Search date:** 2026/4/30. **Number of results:** 1006.

| No. | Search strategies |
| --- | --- |
| #1 | TS=(("ecological momentary assessment" OR "ecologic momentary assessment" OR "experience sampling" OR "experience sampling method*" OR "momentary assessment" OR "momentary data" OR "momentary sampling" OR "ambulatory assessment" OR "real-time assessment" OR "real time assessment" OR "real-time data capture" OR "real time data capture" OR "daily diary" OR "daily diaries" OR "diary assessment" OR "diary method*" OR "electronic diar*" OR "e-diar*" OR "ediar*" OR "computer diar*" OR "digital diar*" OR "mobile diar*" OR "smartphone diar*" OR "event sampling" OR "event-contingent" OR "event contingent" OR "signal-contingent" OR "signal contingent" OR "time-contingent" OR "time contingent" OR "intensive longitudinal" OR "intensive longitudinal assessment*" OR "intensive longitudinal method*" OR "micro-longitudinal" OR microlongitudinal OR (EMA NEAR/3 (assessment* OR sampling OR diar* OR momentary OR ecological)) OR (ESM NEAR/3 (assessment* OR sampling OR method* OR momentary)))) |
| #2 | TS=(neoplasm* OR cancer* OR oncolog* OR tumor* OR tumour* OR carcinoma* OR malignan* OR sarcoma* OR melanoma* OR leukemia* OR leukaemia* OR lymphoma* OR myeloma* OR adenocarcinoma* OR glioma* OR "solid tumor*" OR "solid tumour*" OR "hematologic malignan*" OR "haematologic malignan*" OR "blood cancer*" OR "breast cancer*" OR "breast neoplasm*" OR "lung cancer*" OR "colorectal cancer*" OR "colon cancer*" OR "rectal cancer*" OR "prostate cancer*" OR "gynecologic cancer*" OR "gynaecologic cancer*" OR "ovarian cancer*" OR "cervical cancer*" OR "endometrial cancer*" OR "head and neck cancer*" OR "brain tumor*" OR "brain tumour*" OR "childhood cancer*" OR "pediatric cancer*" OR "paediatric cancer*" OR "cancer survivor*") |
| #3 | #1 AND #2 |

## PubMed

**Search date:** 2026/4/30. **Number of results:** 1277.

| No. | Search strategies |
| --- | --- |
| #1 | "Ecological Momentary Assessment"[MeSH Terms] |
| #2 | ("ecological momentary assessment"[Title/Abstract] OR "ecologic momentary assessment"[Title/Abstract] OR ("EMA"[Title/Abstract] AND (assessment*[Title/Abstract] OR sampling[Title/Abstract] OR diar*[Title/Abstract] OR momentary[Title/Abstract] OR ecological[Title/Abstract])) OR "experience sampling"[Title/Abstract] OR "experience sampling method*"[Title/Abstract] OR ("ESM"[Title/Abstract] AND (assessment*[Title/Abstract] OR sampling[Title/Abstract] OR method*[Title/Abstract] OR momentary[Title/Abstract])) OR "momentary assessment"[Title/Abstract] OR "momentary data"[Title/Abstract] OR "momentary sampling"[Title/Abstract] OR "ambulatory assessment"[Title/Abstract] OR "real-time assessment"[Title/Abstract] OR "real time assessment"[Title/Abstract] OR "real-time data capture"[Title/Abstract] OR "real time data capture"[Title/Abstract] OR "daily diary"[Title/Abstract] OR "daily diaries"[Title/Abstract] OR "diary assessment"[Title/Abstract] OR "diary method*"[Title/Abstract] OR "electronic diar*"[Title/Abstract] OR "e-diar*"[Title/Abstract] OR "ediar*"[Title/Abstract] OR "computer diar*"[Title/Abstract] OR "digital diar*"[Title/Abstract] OR "mobile diar*"[Title/Abstract] OR "smartphone diar*"[Title/Abstract] OR "event sampling"[Title/Abstract] OR "event-contingent"[Title/Abstract] OR "event contingent"[Title/Abstract] OR "signal-contingent"[Title/Abstract] OR "signal contingent"[Title/Abstract] OR "time-contingent"[Title/Abstract] OR "time contingent"[Title/Abstract] OR "intensive longitudinal"[Title/Abstract] OR "intensive longitudinal assessment*"[Title/Abstract] OR "intensive longitudinal method*"[Title/Abstract] OR "micro-longitudinal"[Title/Abstract] OR microlongitudinal[Title/Abstract]) |
| #3 | #1 OR #2 |
| #4 | "Neoplasms"[MeSH Terms] |
| #5 | (neoplasm*[Title/Abstract] OR cancer*[Title/Abstract] OR oncolog*[Title/Abstract] OR tumor*[Title/Abstract] OR tumour*[Title/Abstract] OR carcinoma*[Title/Abstract] OR malignan*[Title/Abstract] OR sarcoma*[Title/Abstract] OR melanoma*[Title/Abstract] OR leukemia*[Title/Abstract] OR leukaemia*[Title/Abstract] OR lymphoma*[Title/Abstract] OR myeloma*[Title/Abstract] OR adenocarcinoma*[Title/Abstract] OR glioma*[Title/Abstract] OR "solid tumor*"[Title/Abstract] OR "solid tumour*"[Title/Abstract] OR "hematologic malignan*"[Title/Abstract] OR "haematologic malignan*"[Title/Abstract] OR "blood cancer*"[Title/Abstract] OR "breast cancer*"[Title/Abstract] OR "breast neoplasm*"[Title/Abstract] OR "lung cancer*"[Title/Abstract] OR "colorectal cancer*"[Title/Abstract] OR "colon cancer*"[Title/Abstract] OR "rectal cancer*"[Title/Abstract] OR "prostate cancer*"[Title/Abstract] OR "gynecologic cancer*"[Title/Abstract] OR "gynaecologic cancer*"[Title/Abstract] OR "ovarian cancer*"[Title/Abstract] OR "cervical cancer*"[Title/Abstract] OR "endometrial cancer*"[Title/Abstract] OR "head and neck cancer*"[Title/Abstract] OR "brain tumor*"[Title/Abstract] OR "brain tumour*"[Title/Abstract] OR "childhood cancer*"[Title/Abstract] OR "pediatric cancer*"[Title/Abstract] OR "paediatric cancer*"[Title/Abstract] OR "cancer survivor*"[Title/Abstract]) |
| #6 | #4 OR #5 |
| #7 | #3 AND #6 |

## Embase

**Search date:** 2026/4/30. **Number of results:** 1846.

| No. | Search strategies |
| --- | --- |
| #1 | "ecological momentary assessment"/exp |
| #2 | (("ecological momentary assessment" OR "ecologic momentary assessment" OR "experience sampling" OR "experience sampling method*" OR "momentary assessment" OR "momentary data" OR "momentary sampling" OR "ambulatory assessment" OR "real-time assessment" OR "real time assessment" OR "real-time data capture" OR "real time data capture" OR "daily diary" OR "daily diaries" OR "diary assessment" OR "diary method*" OR "electronic diar*" OR "e-diar*" OR "ediar*" OR "computer diar*" OR "digital diar*" OR "mobile diar*" OR "smartphone diar*" OR "event sampling" OR "event-contingent" OR "event contingent" OR "signal-contingent" OR "signal contingent" OR "time-contingent" OR "time contingent" OR "intensive longitudinal" OR "intensive longitudinal assessment*" OR "intensive longitudinal method*" OR "micro-longitudinal" OR microlongitudinal OR (EMA NEAR/3 (assessment* OR sampling OR diar* OR momentary OR ecological)) OR (ESM NEAR/3 (assessment* OR sampling OR method* OR momentary)))):ti,ab,kw |
| #3 | #1 OR #2 |
| #4 | "neoplasm"/exp |
| #5 | (neoplasm* OR cancer* OR oncolog* OR tumor* OR tumour* OR carcinoma* OR malignan* OR sarcoma* OR melanoma* OR leukemia* OR leukaemia* OR lymphoma* OR myeloma* OR adenocarcinoma* OR glioma* OR "solid tumor*" OR "solid tumour*" OR "hematologic malignan*" OR "haematologic malignan*" OR "blood cancer*" OR "breast cancer*" OR "breast neoplasm*" OR "lung cancer*" OR "colorectal cancer*" OR "colon cancer*" OR "rectal cancer*" OR "prostate cancer*" OR "gynecologic cancer*" OR "gynaecologic cancer*" OR "ovarian cancer*" OR "cervical cancer*" OR "endometrial cancer*" OR "head and neck cancer*" OR "brain tumor*" OR "brain tumour*" OR "childhood cancer*" OR "pediatric cancer*" OR "paediatric cancer*" OR "cancer survivor*"):ti,ab,kw |
| #6 | #4 OR #5 |
| #7 | #3 AND #6 |

## Cochrane Library

**Search date:** 2026/4/30. **Number of results:** 367.

| No. | Search strategies |
| --- | --- |
| #1 | MeSH descriptor: [Ecological Momentary Assessment] explode all trees |
| #2 | ("ecological momentary assessment" OR "ecologic momentary assessment" OR "experience sampling" OR "experience sampling method" OR "momentary assessment" OR "momentary data" OR "momentary sampling" OR "ambulatory assessment" OR "real-time assessment" OR "real time assessment" OR "real-time data capture" OR "real time data capture" OR "daily diary" OR "daily diaries" OR "diary assessment" OR "diary method" OR "electronic diary" OR "electronic diaries" OR "e-diary" OR "e-diaries" OR "digital diary" OR "digital diaries" OR "computer diary" OR "computer diaries" OR "mobile diary" OR "mobile diaries" OR "smartphone diary" OR "smartphone diaries" OR "event sampling" OR "event-contingent" OR "event contingent" OR "signal-contingent" OR "signal contingent" OR "time-contingent" OR "time contingent" OR "intensive longitudinal" OR "intensive longitudinal assessment" OR "intensive longitudinal method" OR "micro-longitudinal" OR "microlongitudinal" OR (EMA NEAR/3 (assessment OR assessments OR sampling OR diary OR diaries OR momentary OR ecological))):ti,ab,kw |
| #3 | #1 OR #2 |
| #4 | MeSH descriptor: [Neoplasms] explode all trees |
| #5 | (neoplasm* OR cancer* OR oncolog* OR tumor* OR tumour* OR carcinoma* OR malignan* OR sarcoma* OR melanoma* OR leukemia* OR leukaemia* OR lymphoma* OR myeloma* OR adenocarcinoma* OR glioma* OR "solid tumor" OR "solid tumors" OR "solid tumour" OR "solid tumours" OR "hematologic malignancy" OR "hematologic malignancies" OR "haematologic malignancy" OR "haematologic malignancies" OR "blood cancer" OR "blood cancers" OR "breast cancer" OR "breast cancers" OR "breast neoplasm" OR "breast neoplasms" OR "lung cancer" OR "lung cancers" OR "colorectal cancer" OR "colorectal cancers" OR "colon cancer" OR "colon cancers" OR "rectal cancer" OR "rectal cancers" OR "prostate cancer" OR "prostate cancers" OR "gynecologic cancer" OR "gynecologic cancers" OR "gynaecologic cancer" OR "gynaecologic cancers" OR "ovarian cancer" OR "ovarian cancers" OR "cervical cancer" OR "cervical cancers" OR "endometrial cancer" OR "endometrial cancers" OR "head and neck cancer" OR "head and neck cancers" OR "brain tumor" OR "brain tumors" OR "brain tumour" OR "brain tumours" OR "childhood cancer" OR "childhood cancers" OR "pediatric cancer" OR "pediatric cancers" OR "paediatric cancer" OR "paediatric cancers" OR "cancer survivor" OR "cancer survivors"):ti,ab,kw |
| #6 | #4 OR #5 |
| #7 | #3 AND #6 |

## CINAHL

**Search date:** 2026/4/30. **Number of results:** 403.

| No. | Search strategies |
| --- | --- |
| #1 | (MH "Ecological Momentary Assessment") |
| #2 | TI ("ecological momentary assessment" OR "ecologic momentary assessment" OR "experience sampling" OR "experience sampling method*" OR "momentary assessment" OR "momentary data" OR "momentary sampling" OR "ambulatory assessment" OR "real-time assessment" OR "real time assessment" OR "real-time data capture" OR "real time data capture" OR "daily diary" OR "daily diaries" OR "diary assessment" OR "diary method*" OR "electronic diar*" OR "e-diar*" OR "ediar*" OR "computer diar*" OR "digital diar*" OR "mobile diar*" OR "smartphone diar*" OR "event sampling" OR "event-contingent" OR "event contingent" OR "signal-contingent" OR "signal contingent" OR "time-contingent" OR "time contingent" OR "intensive longitudinal" OR "intensive longitudinal assessment*" OR "intensive longitudinal method*" OR "micro-longitudinal" OR microlongitudinal OR (EMA N3 (assessment* OR sampling OR diar* OR momentary OR ecological)) OR (ESM N3 (assessment* OR sampling OR method* OR momentary))) OR AB ("ecological momentary assessment" OR "ecologic momentary assessment" OR "experience sampling" OR "experience sampling method*" OR "momentary assessment" OR "momentary data" OR "momentary sampling" OR "ambulatory assessment" OR "real-time assessment" OR "real time assessment" OR "real-time data capture" OR "real time data capture" OR "daily diary" OR "daily diaries" OR "diary assessment" OR "diary method*" OR "electronic diar*" OR "e-diar*" OR "ediar*" OR "computer diar*" OR "digital diar*" OR "mobile diar*" OR "smartphone diar*" OR "event sampling" OR "event-contingent" OR "event contingent" OR "signal-contingent" OR "signal contingent" OR "time-contingent" OR "time contingent" OR "intensive longitudinal" OR "intensive longitudinal assessment*" OR "intensive longitudinal method*" OR "micro-longitudinal" OR microlongitudinal OR (EMA N3 (assessment* OR sampling OR diar* OR momentary OR ecological)) OR (ESM N3 (assessment* OR sampling OR method* OR momentary))) |
| #3 | #1 OR #2 |
| #4 | (MH "Neoplasms+") |
| #5 | TI (neoplasm* OR cancer* OR oncolog* OR tumor* OR tumour* OR carcinoma* OR malignan* OR sarcoma* OR melanoma* OR leukemia* OR leukaemia* OR lymphoma* OR myeloma* OR adenocarcinoma* OR glioma* OR "solid tumor*" OR "solid tumour*" OR "hematologic malignan*" OR "haematologic malignan*" OR "blood cancer*" OR "breast cancer*" OR "breast neoplasm*" OR "lung cancer*" OR "colorectal cancer*" OR "colon cancer*" OR "rectal cancer*" OR "prostate cancer*" OR "gynecologic cancer*" OR "gynaecologic cancer*" OR "ovarian cancer*" OR "cervical cancer*" OR "endometrial cancer*" OR "head and neck cancer*" OR "brain tumor*" OR "brain tumour*" OR "childhood cancer*" OR "pediatric cancer*" OR "paediatric cancer*" OR "cancer survivor*") OR AB (neoplasm* OR cancer* OR oncolog* OR tumor* OR tumour* OR carcinoma* OR malignan* OR sarcoma* OR melanoma* OR leukemia* OR leukaemia* OR lymphoma* OR myeloma* OR adenocarcinoma* OR glioma* OR "solid tumor*" OR "solid tumour*" OR "hematologic malignan*" OR "haematologic malignan*" OR "blood cancer*" OR "breast cancer*" OR "breast neoplasm*" OR "lung cancer*" OR "colorectal cancer*" OR "colon cancer*" OR "rectal cancer*" OR "prostate cancer*" OR "gynecologic cancer*" OR "gynaecologic cancer*" OR "ovarian cancer*" OR "cervical cancer*" OR "endometrial cancer*" OR "head and neck cancer*" OR "brain tumor*" OR "brain tumour*" OR "childhood cancer*" OR "pediatric cancer*" OR "paediatric cancer*" OR "cancer survivor*") |
| #6 | #4 OR #5 |
| #7 | #3 AND #6 |

## PsycINFO

**Search date:** 2026/4/30. **Number of results:** 15.

| No. | Search strategies |
| --- | --- |
| #1 | DE "Ecological Momentary Assessment" OR DE "Experience Sampling Method" |
| #2 | TI ("ecological momentary assessment" OR "ecologic momentary assessment" OR "experience sampling" OR "experience sampling method*" OR "momentary assessment" OR "momentary data" OR "momentary sampling" OR "ambulatory assessment" OR "real-time assessment" OR "real time assessment" OR "real-time data capture" OR "real time data capture" OR "daily diary" OR "daily diaries" OR "diary assessment" OR "diary method*" OR "electronic diar*" OR "e-diar*" OR "ediar*" OR "computer diar*" OR "digital diar*" OR "mobile diar*" OR "smartphone diar*" OR "event sampling" OR "event-contingent" OR "event contingent" OR "signal-contingent" OR "signal contingent" OR "time-contingent" OR "time contingent" OR "intensive longitudinal" OR "intensive longitudinal assessment*" OR "intensive longitudinal method*" OR "micro-longitudinal" OR microlongitudinal OR (EMA N3 (assessment* OR sampling OR diar* OR momentary OR ecological)) OR (ESM N3 (assessment* OR sampling OR method* OR momentary))) OR AB ("ecological momentary assessment" OR "ecologic momentary assessment" OR "experience sampling" OR "experience sampling method*" OR "momentary assessment" OR "momentary data" OR "momentary sampling" OR "ambulatory assessment" OR "real-time assessment" OR "real time assessment" OR "real-time data capture" OR "real time data capture" OR "daily diary" OR "daily diaries" OR "diary assessment" OR "diary method*" OR "electronic diar*" OR "e-diar*" OR "ediar*" OR "computer diar*" OR "digital diar*" OR "mobile diar*" OR "smartphone diar*" OR "event sampling" OR "event-contingent" OR "event contingent" OR "signal-contingent" OR "signal contingent" OR "time-contingent" OR "time contingent" OR "intensive longitudinal" OR "intensive longitudinal assessment*" OR "intensive longitudinal method*" OR "micro-longitudinal" OR microlongitudinal OR (EMA N3 (assessment* OR sampling OR diar* OR momentary OR ecological)) OR (ESM N3 (assessment* OR sampling OR method* OR momentary))) |
| #3 | #1 OR #2 |
| #4 | DE "Neoplasms" OR DE "Oncology" OR DE "Cancer" |
| #5 | TI (neoplasm* OR cancer* OR oncolog* OR tumor* OR tumour* OR carcinoma* OR malignan* OR sarcoma* OR melanoma* OR leukemia* OR leukaemia* OR lymphoma* OR myeloma* OR adenocarcinoma* OR glioma* OR "solid tumor*" OR "solid tumour*" OR "hematologic malignan*" OR "haematologic malignan*" OR "blood cancer*" OR "breast cancer*" OR "breast neoplasm*" OR "lung cancer*" OR "colorectal cancer*" OR "colon cancer*" OR "rectal cancer*" OR "prostate cancer*" OR "gynecologic cancer*" OR "gynaecologic cancer*" OR "ovarian cancer*" OR "cervical cancer*" OR "endometrial cancer*" OR "head and neck cancer*" OR "brain tumor*" OR "brain tumour*" OR "childhood cancer*" OR "pediatric cancer*" OR "paediatric cancer*" OR "cancer survivor*") OR AB (neoplasm* OR cancer* OR oncolog* OR tumor* OR tumour* OR carcinoma* OR malignan* OR sarcoma* OR melanoma* OR leukemia* OR leukaemia* OR lymphoma* OR myeloma* OR adenocarcinoma* OR glioma* OR "solid tumor*" OR "solid tumour*" OR "hematologic malignan*" OR "haematologic malignan*" OR "blood cancer*" OR "breast cancer*" OR "breast neoplasm*" OR "lung cancer*" OR "colorectal cancer*" OR "colon cancer*" OR "rectal cancer*" OR "prostate cancer*" OR "gynecologic cancer*" OR "gynaecologic cancer*" OR "ovarian cancer*" OR "cervical cancer*" OR "endometrial cancer*" OR "head and neck cancer*" OR "brain tumor*" OR "brain tumour*" OR "childhood cancer*" OR "pediatric cancer*" OR "paediatric cancer*" OR "cancer survivor*") |
| #6 | #4 OR #5 |
| #7 | #3 AND #6 |

## Chinese Databases

**Search date:** 2026/4/30. **Number of results:** CNKI 70, Wanfang 65.

| Database | Search strategies |
| --- | --- |
| China National Knowledge Infrastructure (CNKI) | SU=(生态瞬时评估 + 生态瞬时测量 + 生态瞬时评价 + 经验抽样 + 经验取样 + 经验抽样法 + 体验抽样 + 瞬时评估 + 瞬时测量 + 每日日记 + 日记评估 + 日记法 + 电子日记 + 移动日记 + 实时评估 + 实时监测 + 事件取样 + 信号取样 + 时间取样 + 密集纵向) AND SU=(癌症 + 癌 + 肿瘤 + 恶性肿瘤 + 癌症患者 + 肿瘤患者 + 癌症幸存者 + 白血病 + 淋巴瘤 + 骨髓瘤 + 乳腺癌 + 肺癌 + 胃癌 + 结直肠癌 + 卵巢癌 + 宫颈癌) |
| Wanfang | (标题+摘要+关键词) = (生态瞬时评估 OR 生态瞬时测量 OR 生态瞬时评价 OR 经验抽样 OR 经验取样 OR 经验抽样法 OR 体验抽样 OR 瞬时评估 OR 瞬时测量 OR 每日日记 OR 日记评估 OR 日记法 OR 电子日记 OR 移动日记 OR 实时评估 OR 实时监测 OR 事件取样 OR 信号取样 OR 时间取样 OR 密集纵向) AND (标题+摘要+关键词) = (癌症 OR 癌 OR 肿瘤 OR 恶性肿瘤 OR 癌症患者 OR 肿瘤患者 OR 癌症幸存者 OR 白血病 OR 淋巴瘤 OR 骨髓瘤 OR 乳腺癌 OR 肺癌 OR 胃癌 OR 结直肠癌 OR 卵巢癌 OR 宫颈癌) |
